# Enhancement of Allele Discrimination by Introduction of Nucleotide Mismatches into siRNA in Allele-Specific Gene Silencing by RNAi

**DOI:** 10.1371/journal.pone.0002248

**Published:** 2008-05-21

**Authors:** Yusuke Ohnishi, Yoshiko Tamura, Mariko Yoshida, Katsushi Tokunaga, Hirohiko Hohjoh

**Affiliations:** 1 Department of Molecular Genetics, National Institute of Neuroscience, NCNP, Kodaira, Tokyo, Japan; 2 Department of Human Genetics, Graduate School of Medicine, The University of Tokyo, Tokyo, Japan; University of Sevilla, Spain

## Abstract

Allele-specific gene silencing by RNA interference (RNAi) is therapeutically useful for specifically inhibiting the expression of disease-associated alleles without suppressing the expression of corresponding wild-type alleles. To realize such allele-specific RNAi (ASP-RNAi), the design and assessment of small interfering RNA (siRNA) duplexes conferring ASP-RNAi is vital; however, it is also difficult. In a previous study, we developed an assay system to assess ASP-RNAi with mutant and wild-type reporter alleles encoding the *Photinus* and *Renilla luciferase* genes. In line with experiments using the system, we realized that it is necessary and important to enhance allele discrimination between mutant and corresponding wild-type alleles. Here, we describe the improvement of ASP-RNAi against mutant alleles carrying single nucleotide variations by introducing base substitutions into siRNA sequences, where original variations are present in the central position. Artificially mismatched siRNAs or short-hairpin RNAs (shRNAs) against mutant alleles of the human *Prion Protein* (*PRNP*) gene, which appear to be associated with susceptibility to prion diseases, were examined using this assessment system. The data indicates that introduction of a one-base mismatch into the siRNAs and shRNAs was able to enhance discrimination between the mutant and wild-type alleles. Interestingly, the introduced mismatches that conferred marked improvement in ASP-RNAi, appeared to be largely present in the guide siRNA elements, corresponding to the ‘seed region’ of microRNAs. Due to the essential role of the ‘seed region’ of microRNAs in their association with target RNAs, it is conceivable that disruption of the base-pairing interactions in the corresponding seed region, as well as the central position (involved in cleavage of target RNAs), of guide siRNA elements could influence allele discrimination. In addition, we also suggest that nucleotide mismatches at the 3′-ends of sense-strand siRNA elements, which possibly increase the assembly of antisense-strand (guide) siRNAs into RNA-induced silencing complexes (RISCs), may enhance ASP-RNAi in the case of inert siRNA duplexes. Therefore, the data presented here suggest that structural modification of functional portions of an siRNA duplex by base substitution could greatly influence allele discrimination and gene silencing, thereby contributing to enhancement of ASP-RNAi.

## Introduction

RNA interference (RNAi) is the process of sequence-specific posttranscriptional gene silencing induced by double-stranded RNAs (dsRNAs) homologous to the silenced gene, and it is currently used as a powerful tool to suppress the expression of genes of interest [1], [2]. Introduced or generated dsRNAs are subjected to digestion by an RNase III enzyme, Dicer, into 21–25 nucleotide (nt) RNA duplexes [3]. The resultant RNA duplexes, referred to as small interfering RNA (siRNA) duplexes, are unwound, and single-stranded siRNA elements can then be incorporated into RNA-induced silencing complexes (RISCs) to function as sequence-specific mediators. These are referred to as guide siRNA elements [1], [2], [4].

In mammals, RNAi can be induced by direct introduction of chemically synthesized siRNA duplexes into cells, or by generation of siRNA duplexes using short-hairpin RNA (shRNA) expression vectors. Applications are expanding in various fields of science, with the potential therapeutic use of RNAi in medical science and pharmacogenesis being particularly promising [1], [5]–[8].

Allele-specific gene silencing by RNAi (allele-specific RNAi: ASP-RNAi) is an advanced application of RNAi techniques that allows the expression of an allele of interest to be specifically inhibited [9]. ASP-RNAi would thus be therapeutically very useful, as it can specifically suppress the expression of alleles causing disease without inhibiting the expression of corresponding wild-type alleles [10]–[17]. To induce such ASP-RNAi, it is necessary to design siRNAs that exhibit strong allele-specific gene silencing; thus, siRNAs must be designed such that they are able to carry nucleotide variations characterizing target disease alleles and to discriminate the target alleles from corresponding wild-type alleles. In addition, qualitative and quantitative evaluation of such designed siRNAs on allele-specific gene silencing is required.

In a previous study, we developed an assay system to assess ASP-RNAi with mutant and wild-type reporter alleles encoding the *Photinus* and *Renilla luciferase* genes. In this system, the effects of designed siRNAs and short-hairpin RNAs (shRNAs) against mutant alleles in allele-specific gene silencing, as well as off-target silencing against wild-type alleles, can be simultaneously examined [17]. With amyloid precursor protein (*APP*) variants (the Swedish- and London-type variants) related to familial Alzheimer's disease [18], [19] as model disease alleles, we were able to determine competent siRNA duplexes conferring ASP-RNAi [17]. Previous observations have also suggested that enhanced discrimination of target mutant alleles carrying single nucleotide variations from wild-type allele RNAs is required for ASP-RNAi.

In the present study, we describe the improvement of ASP-RNAi against mutant alleles carrying single nucleotide variations. We introduced base substitutions into siRNA and shRNA sequences, and examined the effects of the resultant mismatched siRNAs and shRNAs on ASP-RNAi by means of our assay system. The results presented here suggest that base substitutions introduced into certain portions of siRNA and shRNA sequences may contribute to enhancement of ASP-RNAi.

## Results

### ASP-RNAi against *PRNP* alleles carrying single nucleotide variations

In a previous study, we established an assessment system for siRNA duplexes conferring ASP-RNAi [17]. This system depends on two reporter alleles encoding the *Photinus* and *Renilla luciferase* genes carrying mutant and wild-type allelic sequences in their 3′-UTRs. Briefly, using this system, the effects of test siRNA duplexes against mutant alleles in allele-specific silencing, as well as off-target silencing against wild-type alleles, can be examined under heterozygous conditions generated by cotransfecting the reporter alleles and siRNA duplexes into cultured human cells.

**Figure 1:**
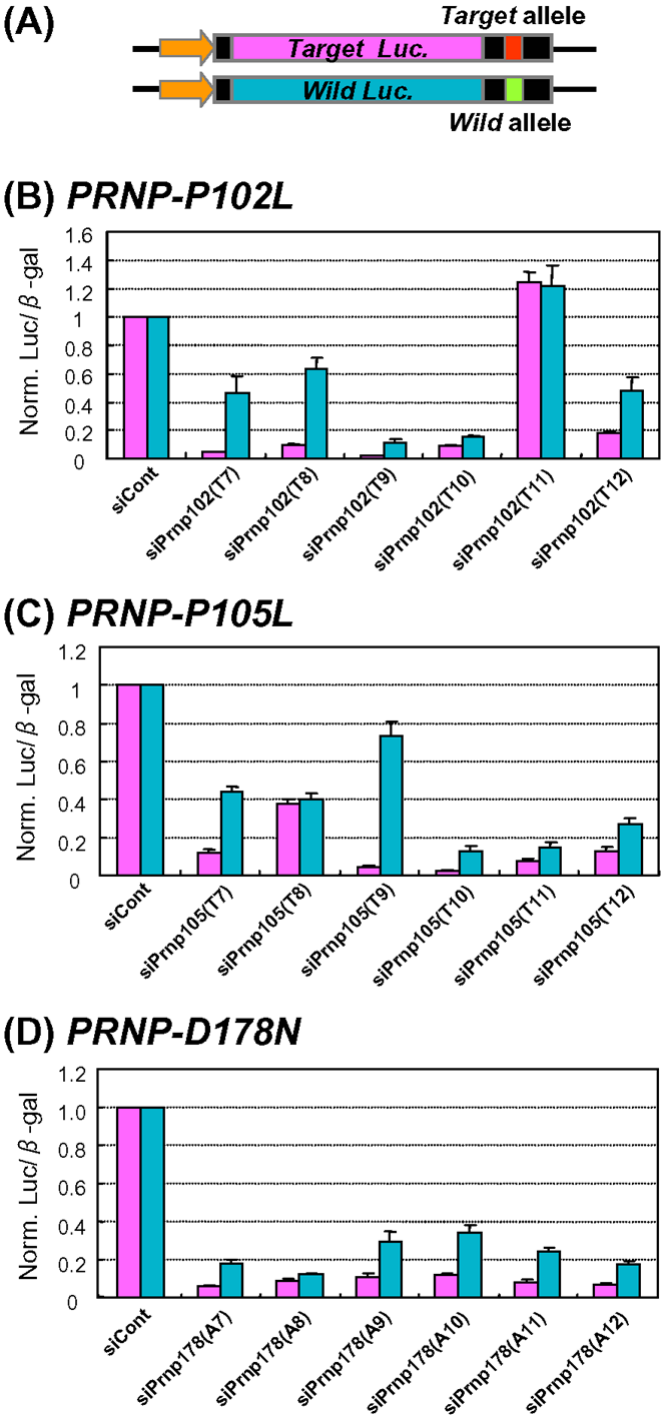
Assessment of ASP-RNAi with reporter alleles. (A) Schematic drawing of reporter alleles. Reporter alleles were constructed by inserting synthetic oligonucleotides of mutant and wild-type allelic sequences into the 3′-UTRs of the reporter genes driven by the same TK promoter (indicated by arrows). Assessment of designed siRNA duplexes against the mutant allele was carried out as described in Materials and Methods. Effects of designed siRNA duplexes against the *PRNP-P102L* (B), *PRNP -P105L* (C), and *PRNP -D178N* (D) mutants on ASP-RNAi. Reporter alleles, synthetic siRNA duplexes against the mutant alleles (indicated) and the *β-galactosidase* gene (control) were cotransfected into HeLa cells. Twenty-four hours after transfection, expression levels of the reporter genes were examined. Levels of either mutant allele (pink boxes) or wild-type allele (blue boxes) luciferase activity were normalized against the levels of β-galactosidase activity, and the ratios of mutant and wild-type luciferase activities in the presence of siRNA duplexes were normalized against the control ratios obtained in the presence of siControl duplex (siCont). Data are averages of at least three independent determinations. Error bars represent standard deviations.

In this study, we focused on the human *Prion Protein (PRNP)* gene, which is known to possess a number of single nucleotide variations [20], [21]. We selected three *PRNP* variants, which are also followed by amino acid substitutions (*P102L, P105L*, and *D178N*) and appear to be associated with susceptibility to various prion diseases such as Gerstmann-Sträussler-Scheinker disease (GSS) and fatal familial insomnia (FFI) [22]–[25]. We constructed three mutant reporter alleles, designated the *PRNP-P102L, PRNP-P105L*, and *PRNP-D178N* alleles, and their corresponding wild-type reporter alleles (Figure 1A). The reporter alleles, synthetic siRNA duplexes against the mutant alleles (supplementary Table s1 and supplementary Figure s1), and the *beta-galactosidase* gene (control), were cotransfected into HeLa cells; thus, the transfected cells were artificially heterozygous with the mutant and wild-type reporter alleles. The effects of the designed siRNA duplexes on suppression of both the mutant and wild-type alleles were then simultaneously examined. As shown in Figure 1, the siRNA duplexes other than siPrnp102(T7), siPrnp102(T8), siPrnp105(T7) and siPrnp105(T9) were not able to induce significant ASP-RNAi. Of the four siRNAs just listed, the siPrnp105(T9) duplex appears to confer ASP-RNAi.

**Figure 2:**
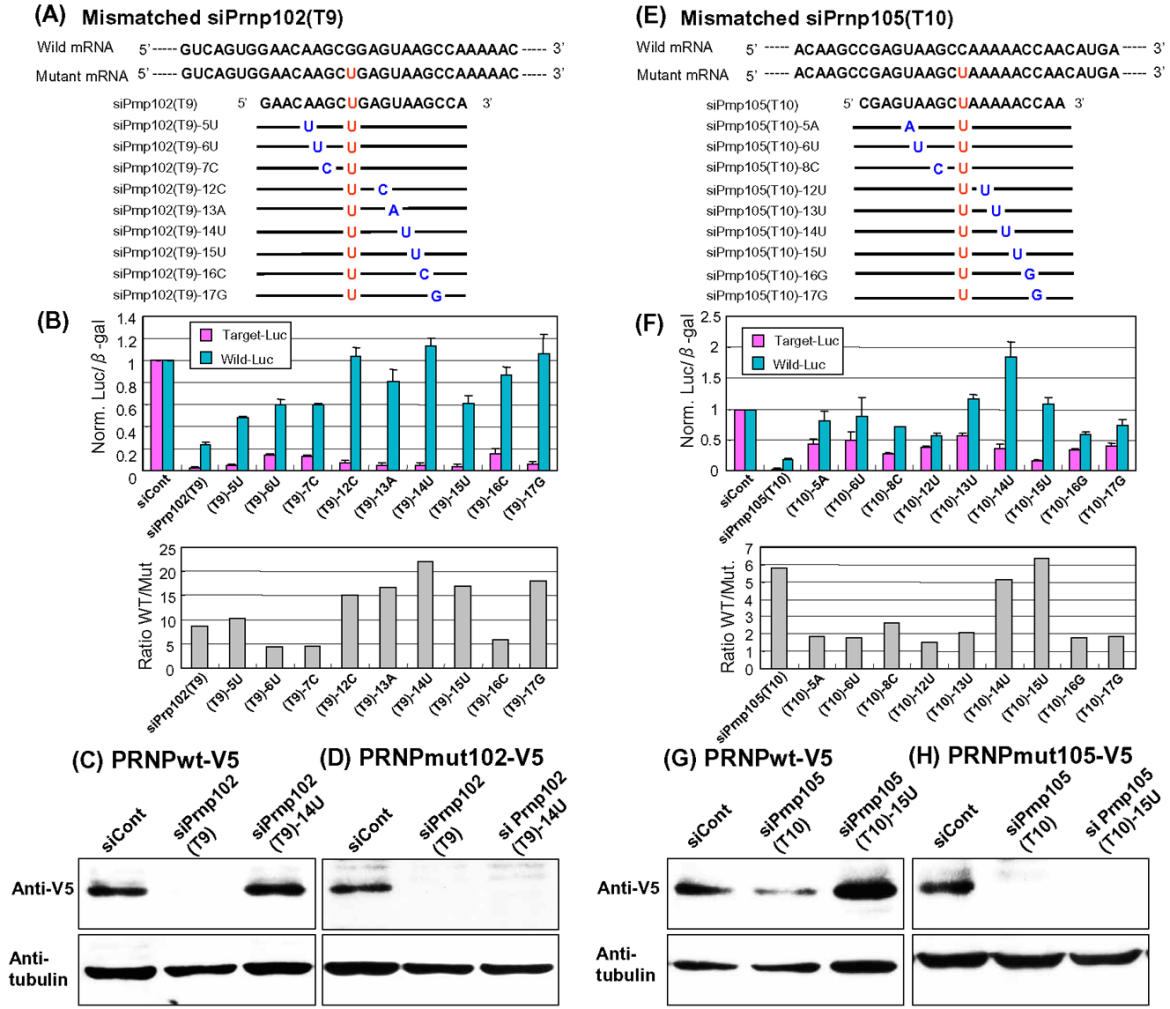
Assessment of mismatched siPrnp102(T9) and siPrnp105(T10) duplexes on ASP-RNAi. (A, E) Nucleotide sequences of wild-type and mutant *PRNP* mRNAs and designed siRNAs. The wild-type and mutant *PRNP* mRNA sequences around the P102L (A) and P105L (E) variations are shown and the variations are indicated in red. Designed siRNAs (indicated) are represented based on the sequence of the sense-strand (passenger) siRNA element; mismatched nucleotides (introduced base substitutions) and the original variations are indicated in blue and red, respectively. The same sequences as the siPrnp102(T9) or siPrnp105(T10) are represented by thin lines. (B, F) Effects of mismatched siPrnp102(T9) and siPrnp105(T10) on ASP-RNAi. Mismatched siPrnp102(T9) (B) and siPrnp105(T10) (E) duplexes (indicated) were examined as in Figure 1. The ratio of wild-type allele-luciferase activity against the mutant allele-luciferase activity (WT/Mut) was also examined to evaluate the improvement in ASP-RNAi. Data are averages of at least three independent determinations. Error bars represent standard deviations. Expression of the wild-type (C, G) and mutant [P102L (D) and P105L (H)] PRNP polypeptides (PRNPmut102-V5 and PRNPmut102-V5) in the presence of indicated siRNA duplexes was investigated by Western blotting using anti-V5 antibody. Expression of α-tubulin was examined as the control.

To realize ASP-RNAi against any target alleles, it is important and necessary to establish techniques for enhancement of allele discrimination followed by specific digestion against the target alleles. To address this, we selected siRNA duplexes possessing strong knockdown potency as candidates for improvement. This is because the only apparent failure of such siRNAs is being unable to discriminate target alleles from non-target ones, i.e., ASP-RNAi may be improved just by reinforcing allele discrimination. Since RNAi activity appears to be influenced by nucleotide mismatches between siRNAs and their target RNAs [11], [26], we introduced a one-base substitution into the selected siRNAs (Figures 2A and E; supplementary Table s2) and examined the effects of such mismatched siRNA duplexes using our assessment system. It should be noted that the resultant mismatched siRNAs give rise to one nucleotide mismatches against the target mutant allele, and more importantly, two nucleotide mismatches against the wild-type allele (the artificially introduced mismatch and the original variation). Accordingly, we expected that such mismatched siRNAs would exhibit better discrimination between target mutant and wild-type alleles, and only suppress the expression of the mutant alleles.

### Effects of mismatched siRNA duplexes on ASP-RNAi against *PRNP-P102L* and *PRNP-P105L* alleles

We selected the siPrnp102(T9) and siPrnp105(T10) duplexes from the first screening of siRNA duplexes against the *PRNP* mutant alleles (Figure 1), and introduced base substitutions into the siRNAs (Figures 2A and E). The resultant mismatched siRNAs were investigated using the assessment system to determine whether they could improve ASP-RNAi. To more precisely determine allele-specific gene silencing, we examined the ratio of the wild-type allele expression to the mutant allele expression. Figures 2B and F show the results of the assessment of the mismatched siPrnp102(T9) and siPrnp105(T10) duplexes, respectively. The results indicate that: (i) introduction of a one-base substitution into the siRNAs was able to influence allele discrimination and RNAi activity, (ii) different nucleotide mismatches appeared to yield different levels of discrimination and inhibition against either the target mutant or wild-type allele, and (iii) some base substitutions appeared to confer marked allele discrimination, resulting in enhancement of ASP-RNAi. It may be of interest that the expression level of the wild-type allele in the presence of siPrnp105(T10)-14U was markedly increased, which may have been caused by the unusual expression level of the control *beta-galactosidase* gene, for as yet unknown reasons. Examination of the ratio of wild-type expression to mutant expression revealed that the siPrnp102(T9)-12C, -13A, -14U and -17G duplexes (Figure 2B) and the siPrnp105(T10)-14U and -15U duplexes (Figure 2F) appear to have markedly improved ASP-RNAi activity. In addition, when HEK293 cells were used instead of HeLa cells, similar results were obtained (data not shown).

To further confirm the results, we examined the effects of the siRNAs on the recognition and inhibition of the *bona fide* wild-type and mutant *PRNP* alleles using their full-length cDNAs. The pPRNPwt-V5 and pPRNPmut102(or mut105)-V5 expression plasmids carrying the wild-type and mutant cDNAs, respectively, were subjected to cotransfection with the siRNAs into HeLa cells, and the expression of the PRNPwt-V5 or the PRNPmut102(or mut105)-V5 polypeptide then examined by Western blotting. The results indicate that while the signal of PRNPwt-V5 in the presence of siPrnp102(T9) or siPrnp105(T10) was reduced, the signal intensity of PRNPwt-V5 was increased in the presence of either siPrnp102(T9)-12C, siPrnp102(T9)-14U or siPrnp105(T10)-15U (Figures 2C and G; supplementary Figure s2A). As expected, the mismatched siRNAs still hold a strong knockdown potency against the mutant *PRNPs* (Figures 2D and H; supplementary Figure s2B). These observations agree with the results for the reporter alleles described above.

### Effects of mismatched siRNA duplexes on ASP-RNAi against *PRNP* mutant alleles

From the first screening (Figures 1C and D), we also selected the siPrnp102(T10) and siPrnp178(A9) duplexes and introduced base substitutions into the siRNAs (supplementary Figures s3A and D). Assessment of the mismatched siRNAs suggested that, similar to Prnp102(T9) and Prnp105(T10), introduction of a one-base substitution into the siRNAs influenced ASP-RNAi activity. Furthermore, the ratios of wild-type allele expression to mutant allele expression indicated that the siPrnp102(T10)-13C and siPrnp178(A9)-13C duplexes have significantly improved ASP-RNAi activity (supplementary Figures s3B and E). Western blot analysis indicated that while the PRNPwt-V5 signal was reduced in the presence of either siPrnp102(T10) or siPrnp178(A9), the signal was increased in the presence of the mismatched siPrnp102(T10)-13C or siPrnp178(A9)-13C duplexes (supplementary Figures s3C and F); thus, the data is also compatible with the results for the reporter alleles described above.

**Figure 3:**
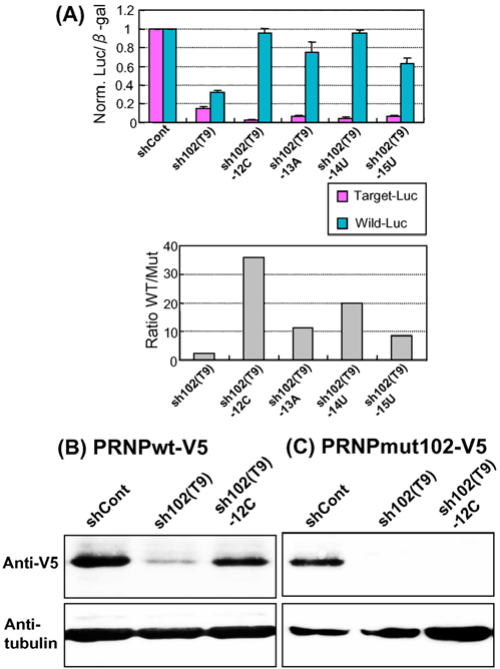
ASP-RNAi with mismatched shRNA expression plasmids against the *PRNP-P102L* mutant. (A) sh102(T9), sh102(T9)-12C, sh102(T9)-14U, sh102(T9)-15U and shCont shRNA expression plasmids for production of siPrnp102(T9), (T9)-12C, (T9)-14U and (T9)-15U duplexes and siControl duplex, respectively, were constructed with reporter alleles and their effects on ASP-RNAi were examined as in Figure 1. Western blot analysis of the wild-type (B) and P102L (C) PRNP polypeptides was carried out as in Figure 2.

### Effects of shRNA expression plasmids on ASP-RNAi

As short-hairpin RNA (shRNA) expression vectors appear to be useful for long-term gene silencing [16], [27], [28], their potential use in ASP-RNAi is also of interest. We constructed shRNA expression plasmids for production of the siPrnp102(T9), (T9)-12C, (T9)-13A, (T9)-14U and (T9)-15U duplexes in cells [designated sh102(T9), sh102(T9)-12C, sh102(T9)-13A, sh102(T9)-14U, sh102(T9)-15U plasmids, respectively]. The shRNA expression plasmids were examined by the assessment system and Western blotting as described above. As shown in Figure 3, the results indicate that while the sh102(T9) plasmid induced strong gene silencing against either the mutant or wild-type allele, the sh102(T9)-12C, -13A, -14U and -15U plasmids were able to confer allele-specific gene silencing, which agrees with the data for synthetic siRNA duplexes (Figure 2). It is noteworthy that the mismatched shRNA expression plasmids appear to enhance ASP-RNAi to a greater degree than the corresponding synthetic siRNA duplexes; of the mismatched plasmids, the sh102(T9)-12C plasmid, appears to induce strong ASP-RNAi. It is possible that mismatched shRNA expression plasmids are superior to mismatched siRNA duplexes for induction of ASP-RNAi.

### Effects of structural modification of siRNA duplexes on ASP-RNAi

In a previous study, we experienced difficulty in inducing ASP-RNAi against the London-type *amyloid precursor protein* (*APP*) mutants, in which single nucleotide substitutions followed by amino acid substitutions (*V717I, V717L, V717G*) were present [17]. While a few designed siRNAs targeting the mutants appeared to discriminate between the mutant and the corresponding wild-type alleles to some degree, most of the siRNAs resulted in weak gene silencing. This is in contrast to the knockdown potency of siRNAs targeting the *PRNP* mutants described above (Figure 1). To improve such ASP-RNAi, it is necessary to design siRNAs such that they can gain knockdown potency against the mutants, and structural modification may also be applicable for achieving such improvements.

**Figure 4:**
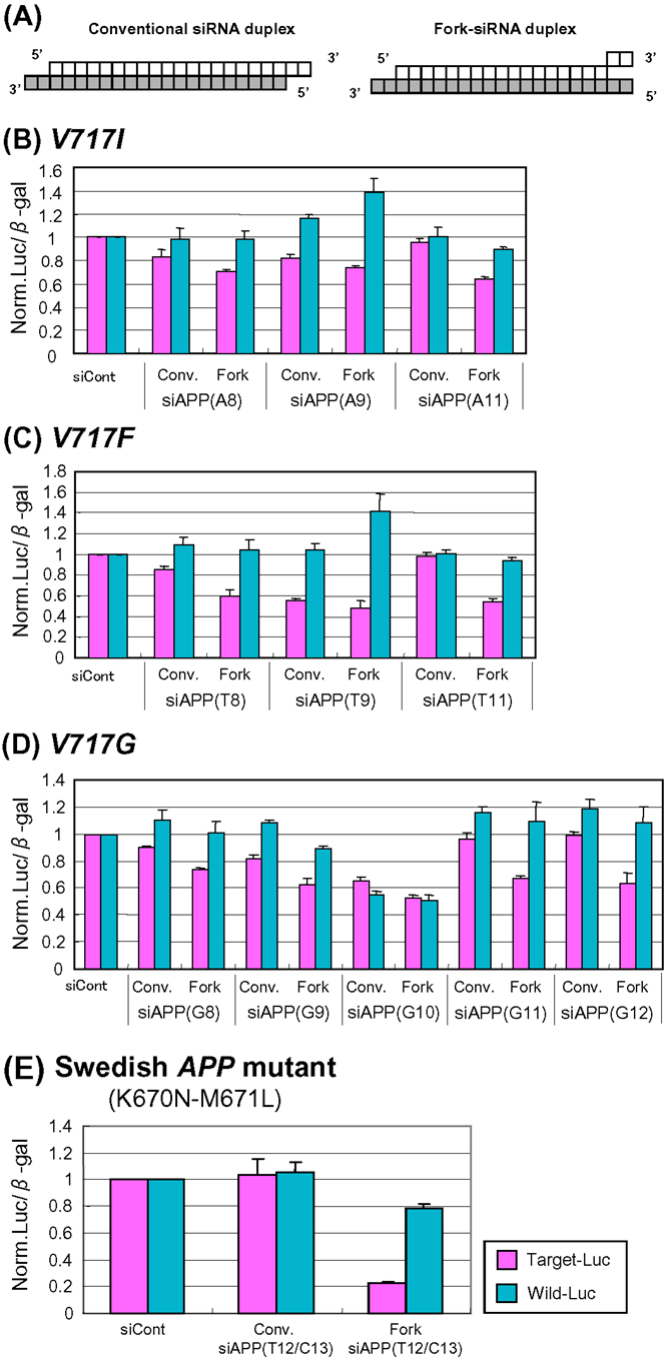
Effects of forked siRNA duplexes on APS-RNAi against *APP* mutants. (A) Schematic drawing of conventional and forked siRNA duplexes. Each box represents a ribonucleotide, and antisense-stranded siRNA elements are indicated by gray boxes. (B)-(E) Comparison of ASP-RNAi activities between conventional (Conv.) and forked (Fork) siRNA duplexes. Assessment of conventional and forked siRNA duplexes against the London *V717I* (B), *V717F* (C), and *V717G* (D) *APP* mutants and the Swedish (E) *APP* mutant was carried out as described previously [17]. The ratios of mutant and wild-type luciferase activities in the presence of siRNA duplexes were normalized against the control ratio obtained in the presence of siControl duplex. Data are averages of at least three independent determinations. Error bars represent standard deviations.

Previous studies showed that fork-siRNA duplexes (F-siRNA duplexes) carrying two nucleotide mismatches at the 3′-ends of the sense-strand siRNA elements are able to enhance RNAi activity to a greater degree than conventional siRNA duplexes [29], [30]. Accordingly, we investigated whether F-siRNA duplexes improve ASP-RNAi activity against the London-type *APP* mutants (Figure 4 and supplementary Table s5). The results indicate that several F-siRNA duplexes [F-siAPP(A11), (T11), (G11) and (G12) duplexes] were able to enhance ASP-RNAi to some degree (Figures 4B-D).

In relation to the improvement of ASP-RNAi against the London-type *APP* mutants, we further observed that F-siRNA duplexes, F-siAPP(T12/C13), targeting the Swedish *APP* mutant carrying double nucleotide mutations, were able to markedly improve ASP-RNAi activity, although the conventional siAPP(T12/C13) duplexes induced little or no RNAi activity (Figure 4E).

### Different effects of miR-196a and miR-196b on recognition of target *HOXB8*

**Figure 5:**
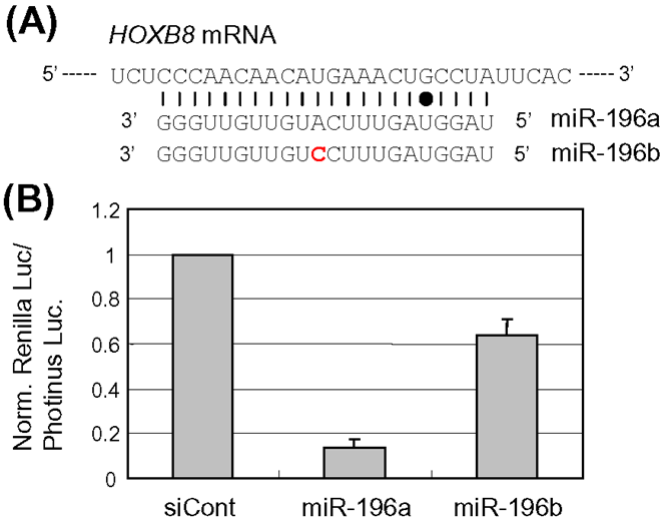
Different knockdown potencies against *HOXB8* between miR-196a and miR-196b. (A) Nucleotide sequences of *HOXB8* mRNA, miR-196a and miR-196b. The *HOXB8* mRNA sequence, which is nearly complementary to miR-196a, together with mature miR-196a and miR-196b are aligned. Perfect base-pairing and G:U wobble base-pairing are indicated by the horizontal bars and dot, respectively. The mismatched base in miR-196b is indicated in red. (B) Effects of miR-196a and miR-196b on gene silencing against *HOXB8*. The miR-196a and miR-196b duplexes were chemically synthesized, as described previously [31]. The synthetic miRNA duplexes, together with a reporter plasmid carrying a part of the *HOXB8* gene (see Materials and Methods), were cotransfected into HeLa cells and the expression of reporter gene was examined. Ratios of normalized target (*Renilla*) luciferase activity to control (*Photinus*) luciferase activity are shown. Data are averages of at least three independent experiments. Error bars represent standard deviations.

From the data of the mismatched siPrnp duplexes (Figures 2 and supplementary Figures s2 and s3) and shPrnp RNAs (Figure 3), it appears that the base substitutions conferring marked improvement are largely present in the region of siRNAs corresponding to the seed region of microRNA. This suggests that the region corresponding to the seed region, as well as the central position (where the original variations are present), of guide siRNAs may play a role in allele discrimination in allele-specific gene silencing (details in Discussion). Based on this, it is of particular interest to determine whether the two regions of *bona fide* microRNA (miRNA) can contribute to the regulation of gene expression. From a previous study [31] and an miRNA database, we focused on miR-196a and miR-196b, both of which are nearly complementary to part of the 3′-UTR sequence of *HOXB8* mRNA. Note that one and two mismatches are present in the predicted base-pairing of *HOXB8* with miR-196a and miR-196b, respectively (Figure 5A). In addition, one mismatch, which can form a G:U wobble base-pair, is present in the seed region of both miRNAs, while the other mismatch (U vs. C) is present in the central position of miR-196b. We examined whether the mismatches in miR-196a and miR-196b participate in the recognition of their target, *HOXB8*.

To address this, we constructed a reporter plasmid encoding the *Renilla luciferase* gene with a part of the *HOXB8* sequence in its 3′-UTR. The resultant reporter gene was cotransfected with either synthetic miR-196a or miR-196b duplex into HeLa cells, and the expression levels of the reporter gene then examined. The results (Figure 5B) indicate that miR-196a induced potent inhibition of the expression of the target reporter gene, whereas miR-196b conferred moderate levels of suppression against target reporter gene expression, thus suggesting different levels of recognition against *HOXB8* between miR-196a and miR-196b. Consequently, the evidence suggests that the mismatches in miR-196a and miR-196b probably influence the recognition of their target *HOXB8* mRNA.

## Discussion

### Enhancement of ASP-RNAi by mismatched siRNAs

**Figure 6:**
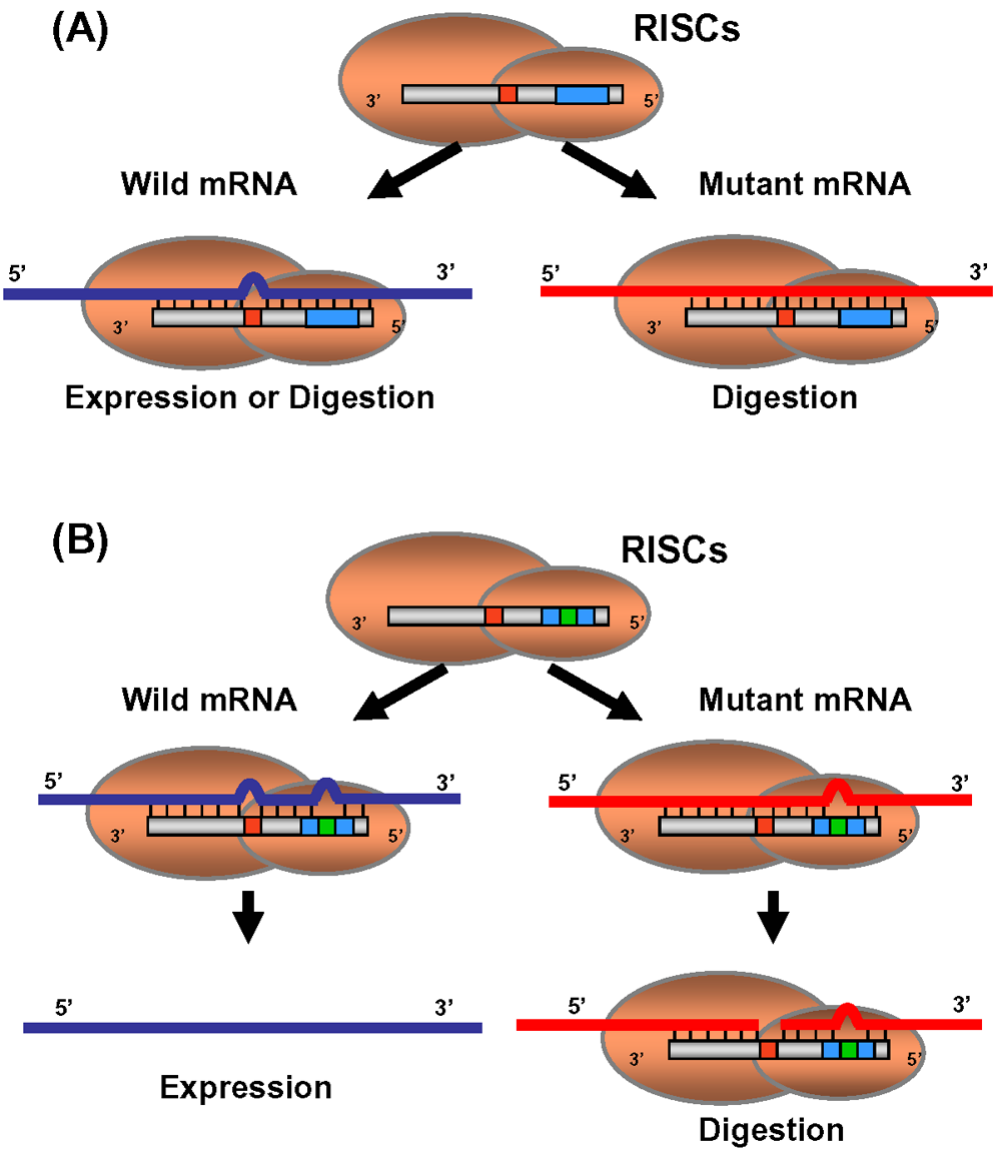
Schematic summary of allele-specific gene silencing with mismatched siRNA. Guide siRNA elements in RISCs are indicated by gray bars, in which nucleotide variation and the seed region are indicated by red and blue boxes, respectively. Introduced base substitutions are indicated by green boxes. Mutant and wild-type allelic transcripts are indicated by red and blue lines, respectively. (A) When designed siRNA targeting the mutant allele has potential for potent gene silencing, not only the mutant but also the wild-type alleles may be inhibited by RNAi mediated by the siRNA, i.e., double knockdown against both alleles may occur. (B) When a one-base substitution is introduced into the seed region of an siRNA conferring double knockdown, the resultant (guide) siRNA generates one and two mismatches against the mutant and wild-type alleles, respectively, thereby possibly gaining the ability to induce ASP-RNAi.

In order to realize and control ASP-RNAi, it is necessary to design competent siRNAs or shRNAs possessing strong allele discrimination between target mutant and wild-type alleles, thereby inducing allele-specific gene silencing. In the case of ASP-RNAi, designed siRNAs perfectly match target mutant alleles, but do not correspond with wild-type alleles, i.e., mismatched base pairing(s) will occur at variation site(s) between the siRNAs and wild-type alleles (Figure 6A; wild-type mRNA). Previous studies in which the effects of single-nucleotide mismatches in siRNAs on RNAi activities were systematically examined suggest that nucleotide mismatches are able to influence RNAi activity [26], [32]. Our present and previous studies indicate that some, but not all, of the designed siRNA duplexes targeting mutant alleles apparently discriminate the mutant alleles from the wild-type alleles. Since different siRNAs confer different levels of RNAi activity depending upon their thermodynamic properties, the intrinsic knockdown potency may also affect allele discrimination between target and non-target alleles.

In the present study, we observed an improvement in ASP-RNAi when siRNAs induced double knockdown of mutant and wild-type alleles: introduction of a one-base substitution into such siRNAs carrying the original variations around the central position appeared to influence allele discrimination and inhibition of target mutant alleles, although different base substitutions conferred different levels of discrimination and inhibition (Figure 6B). This phenomenon may be associated with the thermodynamic properties of the modified siRNA duplexes. Interestingly, the base substitutions conferring marked ASP-RNAi appeared to be largely present in the region of guide siRNAs, corresponding to the seed region of microRNAs. Since such siRNAs exhibit one and two mismatches against mutant and wild-type alleles, respectively, we suggest that disruption of base-pairing interaction in the seed region, as well as the central position, of the guide siRNAs reduces recognition and/or silencing activity against wild-type alleles, and that a one-base mismatch in the seed region of the guide siRNAs against the target mutant alleles hardly affects gene silencing, i.e., potent RNAi activity against the mutant alleles may remain unchanged.

In addition to the improvement in siRNAs conferring double knockdown of mutant and wild-type alleles, we also observed an enhancement of allele discrimination in ASP-RNAi when siRNAs induced weak knockdown potency, which is in contrast to that of the siRNAs described above. In that case, forked siRNA duplexes carrying two nucleotide mismatches at the 3′-ends of the sense-stranded siRNA elements may allow enhanced allele discrimination between target mutant and wild-type alleles. Since forked siRNA duplexes appear to increase the assembly of antisense-strand (guide) siRNA elements into RISCs, the ease of incorporation of antisense-strand siRNA elements into RISCs may be a key factor for improvement of ASP-RNAi activity.

Altogether, the evidence presented here suggests that structural modification by introduction of base substitution into siRNA or shRNA sequences could influence allele discrimination and silencing activity in ASP-RNAi.

### Mismatches in the seed region and the central position of miRNAs affect gene silencing

It is noteworthy that destabilization of base-pairing interaction in the seed region, as well as in the central position, of *bona fide* miRNAs appears to also influence recognition and/or suppression of target RNAs. In the case of miR-196a and miR-196b presented in this study, the miRNAs appear to induce different levels of suppression against *HOXB8* RNA. Accordingly, it is conceivable that the difference in gene silencing involving miRNAs could yield complex regulation of gene expression. Since there are various base-pairing interactions between miRNAs and their target RNAs, such interactions may contribute in the generation of various and complex gene regulatory events. More extensive research is required to further evaluate this possibility.

### Properties of siRNA duplexes conferring ASP-RNAi

Based on the assessed siRNA duplexes efficacy of ASP-RNAi, it appears that different siRNA duplexes induce different levels of ASP-RNAi, suggesting that allele-specific knockdown potencies are most likely dependent on the designed siRNA sequences. Previous studies have suggested that functional siRNA duplexes can be characterized by low base-pairing stability at their 3′-ends [29], [33]–[35], i.e., an asymmetrical feature of base-pairing stability occurs between both ends of functional siRNA duplexes. From the alignment of the siRNA sequences examined in the present and previous studies, it was observed that the siRNA duplexes conferring little or no gene silencing possessed symmetrical base-pairing stability and contained G or C residues at many 3′-ends of their sense-stranded elements (supplementary Table s7A): these features are distinct from those of functional siRNA duplexes. In contrast to the above siRNA duplexes, most of the siRNA duplexes triggering strong RNAi activity, including ASP-RNAi, tended to have asymmetrical base-pairing stability (supplementary Table s7B). It may be of interest to elucidate the properties of siRNA duplexes that confer strong ASP-RNAi, other than the asymmetrical feature. To address this, extensive examination of the knockdown potency of siRNA duplexes targeting various mutant alleles is required.

In conclusion, in order to realize ASP-RNAi against target mutant alleles carrying nucleotide variations, the design and evaluation of competent siRNA duplexes conferring ASP-RNAi is vital; but designed siRNAs do not always confer potent ASP-RNAi activity. The evidence presented here suggests that structural modification of siRNA duplexes by base substitutions may improve ASP-RNAi. The key regions in an siRNA duplex for such modifications are the central position, the seed region and the 3′-end of the sense-strand siRNA element, which appear to be related to target RNA cleavage, target RNA recognition and assembly of the antisense-strand (guide) siRNA element into RISCs, respectively. Therefore, structural modification of such functional portions of siRNA duplexes may greatly influence allele discrimination and gene silencing activity, thereby conferring improvement of ASP-RNAi.

## Materials and Methods

### Preparation of oligonucleotides

RNA and DNA oligonucleotides were obtained from TAKARA BIO, and INVITROGEN or BEX, respectively. For preparation of duplexes, sense- and antisense-strand oligonucleotides (20 µM each) were mixed and annealed, as described previously [29]. Sequences of synthesized RNA and DNA oligonucleotides are shown in supplementary Tables s1, s2, s3, s4, s5 and s6. Non-silencing siRNA duplex (siControl; Qiagen) was used as the negative control.

### Cell culture

HeLa and HEK293 cells were grown at 37°C in Dulbecco's modified Eagle's medium (Wako) supplemented with 10% fetal bovine serum (Sigma), 100 U/ml penicillin and 100 µg/ml streptomycin (Wako) in a 5% CO_2_-humidified chamber. HEK293 cells (Registry No. JCRB9068) were obtained from the Health Science Research Resources Bank.

### Construction of reporter alleles and shRNA expression plasmids

Reporter alleles were constructed as described previously [17]. Briefly, phRL-TK (Promega) and pGL3-TK [30] plasmids encoding the *Renilla* and *Photinus luciferase* genes, respectively, were digested with XbaΙ and NotΙ, and subjected to ligation with synthetic oligonucleotide duplexes corresponding to the *PRNP-P102L, PRNP-P105L* and *PRNP-D178N* alleles, as well as wild-type alleles (oligonucleotide sequences are indicated in supplementary Table s3). The resultant plasmids carry allelic *PRNP* sequences in the 3′-untranslated regions (UTRs) of the *luciferase* genes (Figure 1A). The London-type *APP* mutant and wild-type *APP* reporter alleles described in a previous paper [17] were also used. For construction of shRNA expression vectors, the GeneSilencer shRNA Vector (Gene Therapy System, Inc.) was used, and synthetic oligonucleotide duplexes were inserted into the vector according to the manufacturer's instructions (oligonucleotide sequences are indicated in Table s4). To construct a reporter plasmid carrying part of the *HOXB8* sequence, psiCHECK-2 vector (Promega) was digested with XhoΙ and PmeΙ, and subjected to ligation with synthetic *HOXB8* oligonucleotide duplex (supplementary Table s3). With regard to expression plasmids carrying a full-length cDNA of the human *PRNP* linked with the V5-tag sequence, the wild-type *PRNP* cDNA was amplified by RT-PCR with human brain total RNA (Ambion), trimmed with EcoRΙ and NotΙ, and inserted into the pTracer-EF/Bsd vector (Invitrogen); the resultant plasmid was designated ‘pPRNPwt-V5’. Using this plasmid, mutant plasmids carrying the *PRNP P102L* and *P105L* were constructed using the GeneEditor *in vitro* Site-Directed Mutagenesis System (Promega) and the QuikChange Site-Directed Mutagenesis Kit (Stratagene), respectively, according to the manufacturer's instructions; the resultant mutant expression plasmids were designated ‘pPRNPmut102-V5’ and ‘pPRNPmut105-V5’. The PCR primers for *PRNP* cDNA synthesis and oligonucleotides used for the mutagenesis were as follows:

For *PRNP* cDNA synthesis;

Forward PCR primer; 5′-TTCCGAATTCGCCACCATGGCGAACCTTGGCTGCT-3′

Reverse PCR primer; 5′-ACATTGCGGCCGCTCCCACTATCAGGAAGATGAGG-3′

For site-directed mutagenesis:

*PRNP P102L* oligo DNA;

5′-pGTGGAACAAGCTGAGTAAGCCAA-3′

*PRNP P105L* oligo DNAs;

Forward;

5′-GGAACAAGCCGAGTAAGCTAAAAACCAACATGAAGCACATGGC-3′

Reverse;

5′- GCCATGTGCTTCATGTTGGTTTTTAGCTTACTCGGCTTGTTCC-3′

### Transfection and reporter assay

The day before transfection, cells were trypsinized, diluted with fresh medium without antibiotics, and seeded into 24-well culture plates (approximately 0.5×10^5^ cells/well). Next, 0.24 µg (40 nM) of siRNA duplexes or 0.1 µg of shRNA vectors together with 0.2 µg of pGL3-TK-backbone plasmid, 0.05 µg of phRL-TK-backbone plasmid and 0.1 µg of pSV-β-Galactosidase control vector (Promega) as a control were applied to each well using Lipofectamine 2000 transfection reagent (Invitrogen) as described previously [30]. Twenty-four hours after transfection, cell lysate was prepared and expression levels of luciferase and β-galactosidase were examined using the Dual-Luciferase reporter assay system (Promega) and Beta-Glo assay system (Promega), respectively, according to the manufacturer's instructions. The luminescent signals were measured using a TD-20/20 luminometer (Promega).

### Western blotting

The pPRNPwt-V5 or pPRNPmut102(or mut105)-V5 plasmid (0.1 µg) was cotransfected with siRNA duplex (0.24 µg) or shRNA expression plasmid (0.1 µg) into HeLa cells. Forty-eight hours after transfection, cell lysate was prepared and examined by Western blotting. Equal amounts of cell lysate were separated by SDS-PAGE and electrophoretically blotted onto PVDF membranes (Millipore). Membranes were blocked for 1 h in blocking solution [5% non-fat milk in washing buffer (0.1% Tween-20 in PBS)] and incubated with anti-V5 antibody (Invitrogen) or anti-α-tubulin antibody DM1A (Sigma), followed by washing in PBS containing 0.1% Tween-20 and further incubation with horseradish peroxidase-conjugated donkey anti-mouse IgG (Jackson ImmunoResearch Laboratories). Antigen-antibody complexes were visualized using Immobilon Western reagent (Millipore). The experiments were duplicated at least twice independently.

## Supporting Information

Figure S1 Wild-type and mutant PRNP mRNAs and designed sense-strand siRNAs. The wild-type and mutant PRNP mRNA sequences around the P102L (A), P105L (B), and D178N (C) variations, respectively, are shown and the nucleotide variations are indicated in red. Designed siRNAs (indicated) are represented by thin lines and only the variations are indicated in red.
(0.26 MB TIF)

Figure S2 Expression of the wild-type (A) and P102L (B) PRNP polypeptides in the presence of indicated siRNAs was examined by Western blotting as in Figure 2. Expression of alpha-tubulin was also examined as the control.
(0.99 MB TIF)

Figure S3 Assessment of mismatched siPrnp102(T10) and siPrnp178(A9) on ASP-RNAi. (A, D) Nucleotide sequences of wild-type and mutant PRNP mRNAs and designed siRNAs are indicated as in Figure 2. (B, E) Assessment of mismatched siRNAs was carried out and the results are shown as in Figure 2. (C, F) Western blot analysis of the wild-type PRNP polypeptide (PRNPwt-V5) in the presence of indicated siRNAs was carried out as in Figure 2. The PRNPwt-V5 gene was driven by the EF-1α promoter instead of the CMV promoter in this experiment. Expression of α-tubulin was also examined as the control.
(0.61 MB TIF)

Table S1 (0.04 MB DOC)

Table S2 (0.05 MB DOC)

Table S3 (0.03 MB DOC)

Table S4 (0.03 MB DOC)

Table S5 (0.04 MB DOC)

Table S6 (0.03 MB DOC)

Table S7 (0.11 MB DOC)

## References

[1] Dykxhoorn DM, Novina CD, Sharp PA (2003). Killing the messenger: short RNAs that silence gene expression.. Nat Rev Mol Cell Biol.

[2] Meister G, Tuschl T (2004). Mechanisms of gene silencing by double-stranded RNA.. Nature.

[3] Bernstein E, Caudy AA, Hammond SM, Hannon GJ (2001). Role for a bidentate ribonuclease in the initiation step of RNA interference.. Nature.

[4] Matranga C, Tomari Y, Shin C, Bartel DP, Zamore PD (2005). Passenger-strand cleavage facilitates assembly of siRNA into Ago2-containing RNAi enzyme complexes.. Cell.

[5] Paddison PJ, Silva JM, Conklin DS, Schlabach M, Li M (2004). A resource for large-scale RNA-interference-based screens in mammals.. Nature.

[6] Caplen NJ (2004). Gene therapy progress and prospects. Downregulating gene expression: the impact of RNA interference.. Gene Ther.

[7] Karagiannis TC, El-Osta A (2005). RNA interference and potential therapeutic applications of short interfering RNAs.. Cancer Gene Ther.

[8] Bonini NM, La Spada AR (2005). Silencing polyglutamine degeneration with RNAi.. Neuron.

[9] Victor M, Bei Y, Gay F, Calvo D, Mello C (2002). HAT activity is essential for CBP-1-dependent transcription and differentiation in Caenorhabditis elegans.. EMBO Rep.

[10] Wood M, Yin H, McClorey G (2007). Modulating the expression of disease genes with RNA-based therapy.. PLoS Genet.

[11] Miller VM, Xia H, Marrs GL, Gouvion CM, Lee G (2003). Allele-specific silencing of dominant disease genes.. Proc Natl Acad Sci U S A.

[12] Miller VM, Gouvion CM, Davidson BL, Paulson HL (2004). Targeting Alzheimer's disease genes with RNA interference: an efficient strategy for silencing mutant alleles.. Nucleic Acids Res.

[13] Rodriguez-Lebron E, Paulson HL (2006). Allele-specific RNA interference for neurological disease.. Gene Ther.

[14] Maxwell MM, Pasinelli P, Kazantsev AG, Brown RH (2004). RNA interference-mediated silencing of mutant superoxide dismutase rescues cyclosporin A-induced death in cultured neuroblastoma cells.. Proc Natl Acad Sci U S A.

[15] Denovan-Wright EM, Davidson BL (2006). RNAi: a potential therapy for the dominantly inherited nucleotide repeat diseases.. Gene Ther.

[16] Xia H, Mao Q, Eliason SL, Harper SQ, Martins IH (2004). RNAi suppresses polyglutamine-induced neurodegeneration in a model of spinocerebellar ataxia.. Nat Med.

[17] Ohnishi Y, Tokunaga K, Kaneko K, Hohjoh H (2006). Assessment of allele-specific gene silencing by RNA interference with mutant and wild-type reporter alleles.. Journal of RNAi and Gene Silencing.

[18] Mullan M, Crawford F, Axelman K, Houlden H, Lilius L (1992). A pathogenic mutation for probable Alzheimer's disease in the APP gene at the N-terminus of beta-amyloid.. Nat Genet.

[19] Goate A, Chartier-Harlin MC, Mullan M, Brown J, Crawford F (1991). Segregation of a missense mutation in the amyloid precursor protein gene with familial Alzheimer's disease.. Nature.

[20] Collinge J (1997). Human prion diseases and bovine spongiform encephalopathy (BSE).. Hum Mol Genet.

[21] Jackson GS, Collinge J (2001). The molecular pathology of CJD: old and new variants.. Mol Pathol.

[22] Prusiner SB (1998). Prions.. Proc Natl Acad Sci U S A.

[23] Hsiao K, Baker HF, Crow TJ, Poulter M, Owen F (1989). Linkage of a prion protein missense variant to Gerstmann-Straussler syndrome.. Nature.

[24] Yamada M, Itoh Y, Inaba A, Wada Y, Takashima M (1999). An inherited prion disease with a PrP P105L mutation: clinicopathologic and PrP heterogeneity.. Neurology.

[25] Chen SG, Parchi P, Brown P, Capellari S, Zou W (1997). Allelic origin of the abnormal prion protein isoform in familial prion diseases.. Nat Med.

[26] Du Q, Thonberg H, Wang J, Wahlestedt C, Liang Z (2005). A systematic analysis of the silencing effects of an active siRNA at all single-nucleotide mismatched target sites.. Nucleic Acids Res.

[27] Raoul C, Abbas-Terki T, Bensadoun JC, Guillot S, Haase G (2005). Lentiviral-mediated silencing of SOD1 through RNA interference retards disease onset and progression in a mouse model of ALS.. Nat Med.

[28] Singer O, Marr RA, Rockenstein E, Crews L, Coufal NG (2005). Targeting BACE1 with siRNAs ameliorates Alzheimer disease neuropathology in a transgenic model.. Nat Neurosci.

[29] Hohjoh H (2004). Enhancement of RNAi activity by improved siRNA duplexes.. FEBS Lett.

[30] Ohnishi Y, Tokunaga K, Hohjoh H (2005). Influence of assembly of siRNA elements into RNA-induced silencing complex by fork-siRNA duplex carrying nucleotide mismatches at the 3′- or 5′-end of the sense-stranded siRNA element.. Biochem Biophys Res Commun.

[31] Yekta S, Shih IH, Bartel DP (2004). MicroRNA-directed cleavage of HOXB8 mRNA.. Science.

[32] Schwarz DS, Ding H, Kennington L, Moore JT, Schelter J (2006). Designing siRNA that distinguish between genes that differ by a single nucleotide.. PLoS Genet.

[33] Ui-Tei K, Naito Y, Takahashi F, Haraguchi T, Ohki-Hamazaki H (2004). Guidelines for the selection of highly effective siRNA sequences for mammalian and chick RNA interference.. Nucleic Acids Res.

[34] Schwarz DS, Hutvagner G, Du T, Xu Z, Aronin N (2003). Asymmetry in the assembly of the RNAi enzyme complex.. Cell.

[35] Khvorova A, Reynolds A, Jayasena SD (2003). Functional siRNAs and miRNAs exhibit strand bias.. Cell.

