# Supplementary figures and images for: Enhancement of Allele Discrimination by Introduction of Nucleotide Mismatches into siRNA in Allele-Specific Gene Silencing by RNAi
Source: PLoS One. 2008 May 21;3(5):e2248. doi: 10.1371/journal.pone.0002248 (PMC2373929; doi:10.1371/journal.pone.0002248)

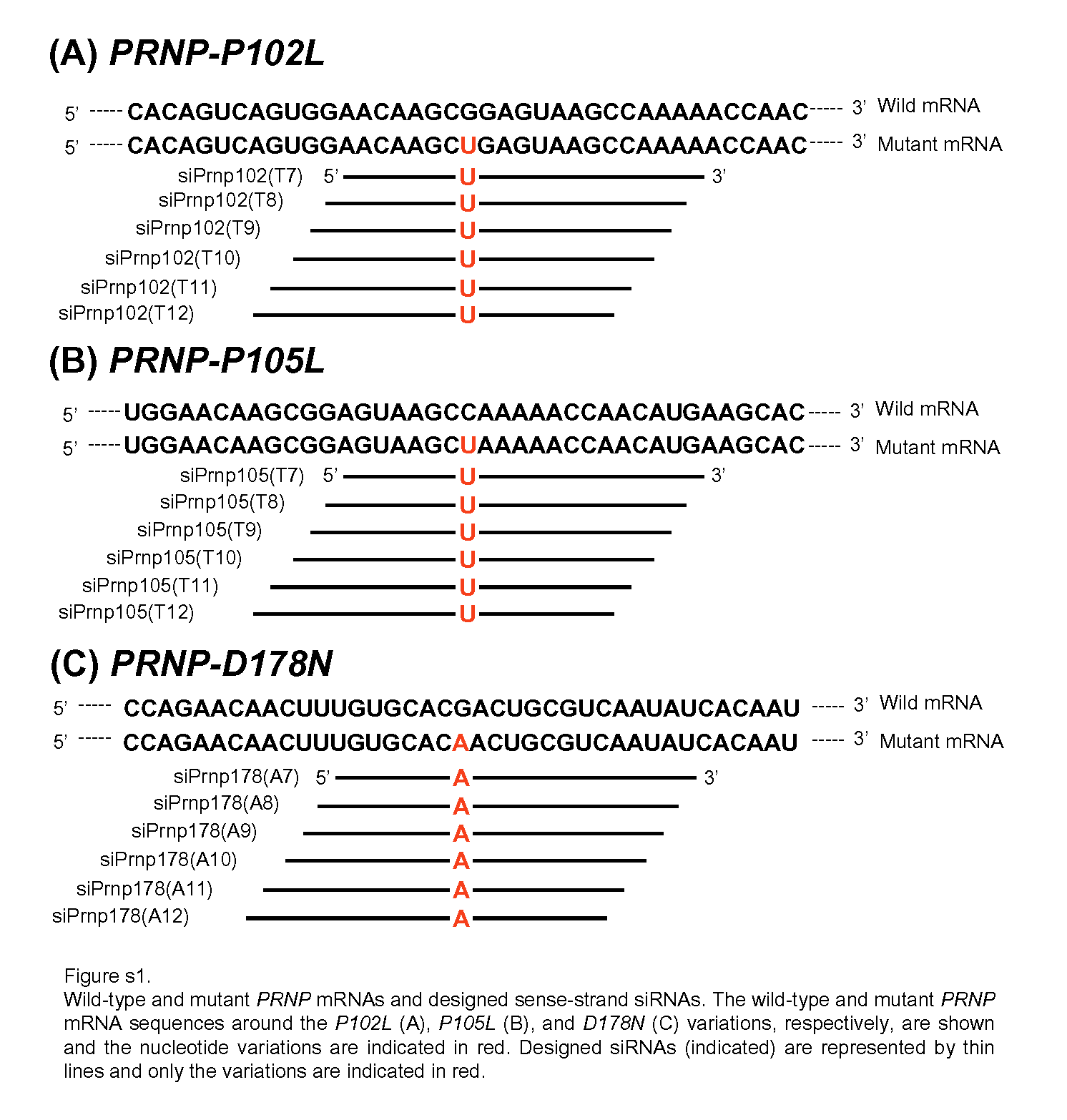

Supplement: Figure S1 — Wild-type and mutant PRNP mRNAs and designed sense-strand siRNAs. The wild-type and mutant PRNP mRNA sequences around the P102L (A), P105L (B), and D178N (C) variations, respectively, are shown and the nucleotide variations are indicated in red. Designed siRNAs (indicated) are represented by thin lines and only the variations are indicated in red. (0.26 MB TIF) [file pone.0002248.s001.tif]

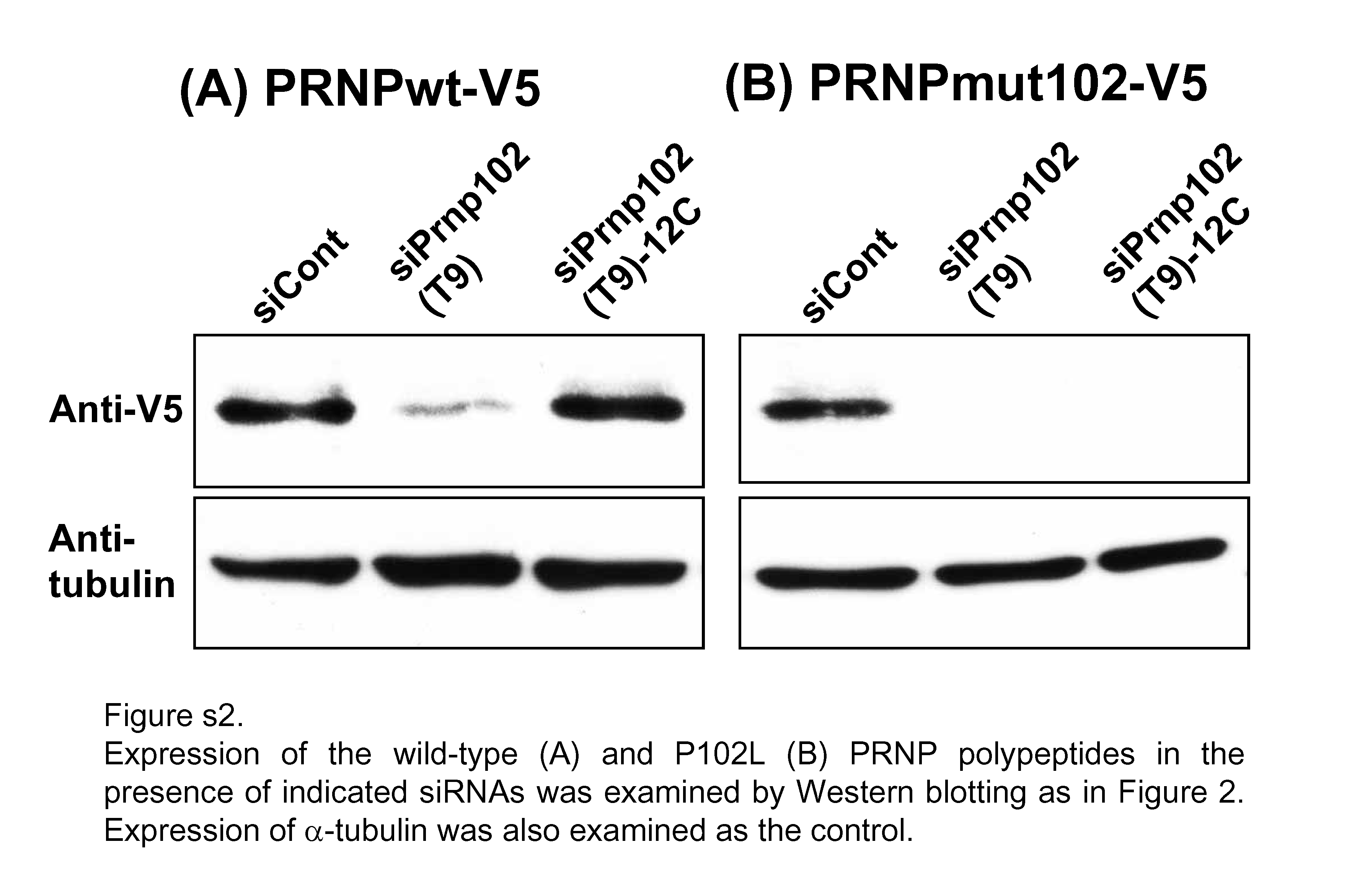

Supplement: Figure S2 — Expression of the wild-type (A) and P102L (B) PRNP polypeptides in the presence of indicated siRNAs was examined by Western blotting as in Figure 2. Expression of alpha-tubulin was also examined as the control. (0.99 MB TIF) [file pone.0002248.s002.tif]

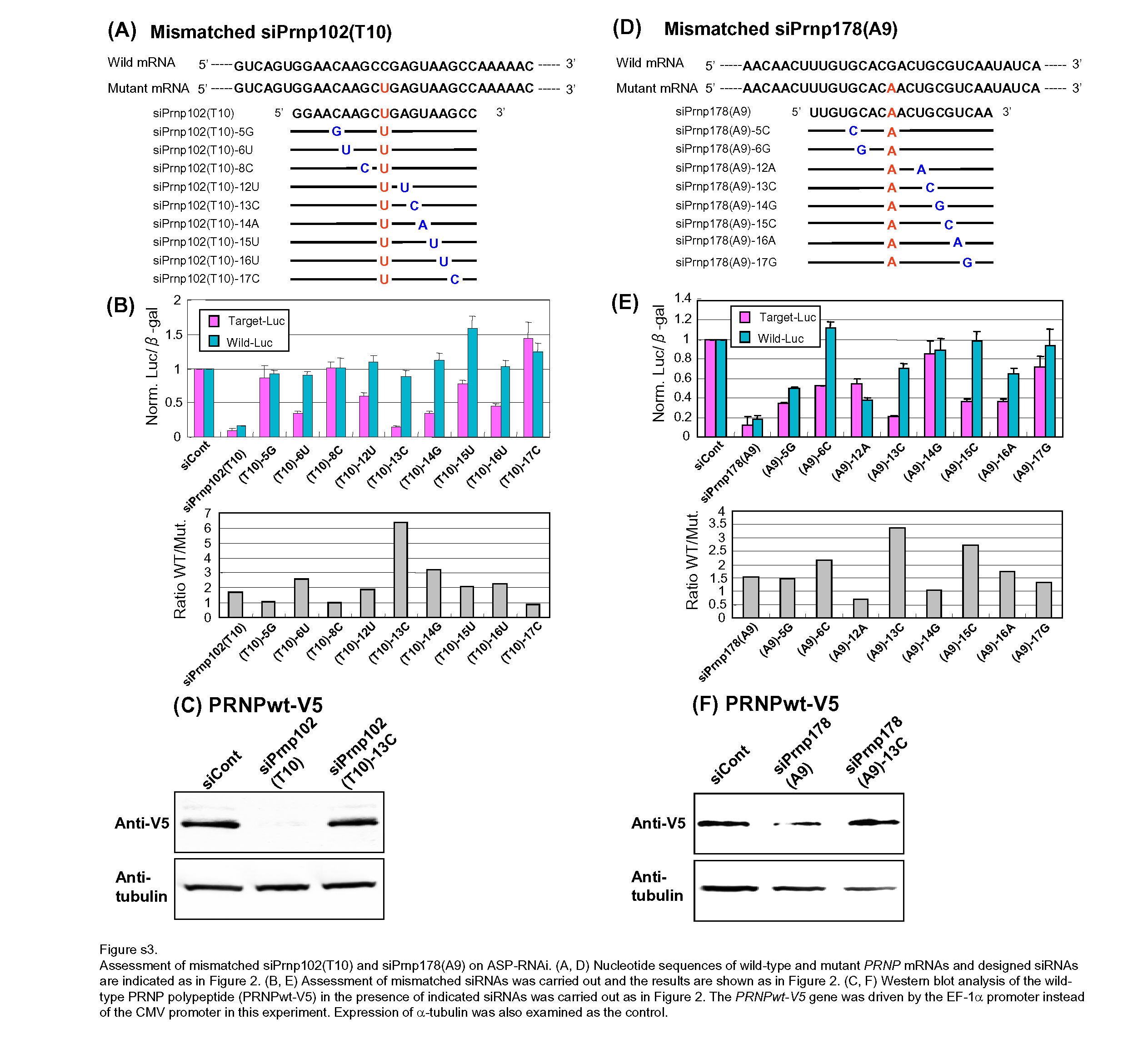

Supplement: Figure S3 — Assessment of mismatched siPrnp102(T10) and siPrnp178(A9) on ASP-RNAi. (A, D) Nucleotide sequences of wild-type and mutant PRNP mRNAs and designed siRNAs are indicated as in Figure 2. (B, E) Assessment of mismatched siRNAs was carried out and the results are shown as in Figure 2. (C, F) Western blot analysis of the wild-type PRNP polypeptide (PRNPwt-V5) in the presence of indicated siRNAs was carried out as in Figure 2. The PRNPwt-V5 gene was driven by the EF-1α promoter instead of the CMV promoter in this experiment. Expression of α-tubulin was also examined as the control. (0.61 MB TIF) [file pone.0002248.s003.tif]
