# Supplementary material for: Enhancement of Allele Discrimination by Introduction of Nucleotide Mismatches into siRNA in Allele-Specific Gene Silencing by RNAi
Source: PLoS One. 2008 May 21;3(5):e2248. doi: 10.1371/journal.pone.0002248 (PMC2373929; doi:10.1371/journal.pone.0002248)
Supplement: Table S1 — (0.04 MB DOC) [file pone.0002248.s004.doc]

Table s1. Synthetic siRNAs against the *PRNP* variants

| Name | Seq. (5’---------------------3’) |
| --- | --- |
| siPrnp102(T7) | ACAAGC**U**GAGUAAGCCAAAUU |
| siPrnp102(T8) | AACAAGC**U**GAGUAAGCCAAUU |
| siPrnp102(T9) | GAACAAGC**U**GAGUAAGCCAUU |
| siPrnp102(T10) | GGAACAAGC**U**GAGUAAGCCUU |
| siPrnp102(T11) | UGGAACAAGC**U**GAGUAAGCUU |
| siPrnp102(T12) | GUGGAACAAGC**U**GAGUAAGUU |
| siPrnp105(T7) | GUAAGC**U**AAAAACCAACAUUU |
| siPrnp105(T8) | AGUAAGC**U**AAAAACCAACAUU |
| siPrnp105(T9) | GAGUAAGC**U**AAAAACCAACUU |
| siPrnp105(T10) | CGAGUAAGC**U**AAAAACCAAUU |
| siPrnp105(T11) | CCGAGUAAGC**U**AAAAACCAUU |
| siPrnp105(T12) | GCCGAGUAAGC**U**AAAAACCUU |
| siPrnp178(A7) | GUGCAC**A**ACUGCGUCAAUAUU |
| siPrnp178(A8) | UGUGCAC**A**ACUGCGUCAAUUU |
| siPrnp178(A9) | UUGUGCAC**A**ACUGCGUCAAUU |
| siPrnp178(A10) | UUUGUGCAC**A**ACUGCGUCAUU |
| siPrnp178(A11) | CUUUGUGCAC**A**ACUGCGUCUU |
| siPrnp178(A12) | ACUUUGUGCAC**A**ACUGCGUUU |

Sense (passenger)-strand siRNAs containing 2-nt ribo-uridine (UU) 3’ overhangs are indicated.

Variation sites are indicated in red.
