# Supplementary material for: Enhancement of Allele Discrimination by Introduction of Nucleotide Mismatches into siRNA in Allele-Specific Gene Silencing by RNAi
Source: PLoS One. 2008 May 21;3(5):e2248. doi: 10.1371/journal.pone.0002248 (PMC2373929; doi:10.1371/journal.pone.0002248)
Supplement: Table S2 — (0.05 MB DOC) [file pone.0002248.s005.doc]

Table s2. Synthetic siRNAs including mismatched nucleotides

| Name | Seq. (5’---------------------3’) |
| --- | --- |
| siPrnp102(T9)-5U | GAAC**U**AGCUGAGUAAGCCAUU |
| siPrnp102(T9)-6U | GAACA**U**GCUGAGUAAGCCAUU |
| siPrnp102(T9)-7C | GAACAA**C**CUGAGUAAGCCAUU |
| siPrnp102(T9)-12C | GAACAAGCUGA**C**UAAGCCAUU |
| siPrnp102(T9)-13A | GAACAAGCUGAG**A**AAGCCAUU |
| siPrnp102(T9)-14U | GAACAAGCUGAGU**U**AGCCAUU |
| siPrnp102(T9)-15U | GAACAAGCUGAGUA**U**GCCAUU |
| siPrnp102(T9)-16C | GAACAAGCUGAGUAA**C**CCAUU |
| siPrnp102(T9)-17G | GAACAAGCUGAGUAAG**G**CAUU |
| siPrnp102(T10)-5G | GGAA**G**AAGCUGAGUAAGCCUU |
| siPrnp102(T10)-6U | GGAAC**U**AGCUGAGUAAGCCUU |
| siPrnp102(T10)-8C | GGAACAA**C**CUGAGUAAGCCUU |
| siPrnp102(T10)-12U | GGAACAAGCUG**U**GUAAGCCUU |
| siPrnp102(T10)-13C | GGAACAAGCUGA**C**UAAGCCUU |
| siPrnp102(T10)-14A | GGAACAAGCUGAG**A**AAGCCUU |
| siPrnp102(T10)-15U | GGAACAAGCUGAGU**U**AGCCUU |
| siPrnp102(T10)-16U | GGAACAAGCUGAGUA**U**GCCUU |
| siPrnp102(T10)-17C | GGAACAAGCUGAGUAA**C**CCUU |
| siPrnp105(T10)-5A | CGAG**A**AAGCUAAAAACCAAUU |
| siPrnp105(T10)-6U | CGAGU**U**AGCUAAAAACCAAUU |
| siPrnp105(T10)-8C | CGAGUAA**C**CUAAAAACCAAUU |
| siPrnp105(T10)-12U | CGAGUAAGCUA**U**AAACCAAUU |
| siPrnp105(T10)-13U | CGAGUAAGCUAA**U**AACCAAUU |
| siPrnp105(T10)-14U | CGAGUAAGCUAAA**U**ACCAAUU |
| siPrnp105(T10)-15U | CGAGUAAGCUAAAA**U**CCAAUU |
| siPrnp105(T10)-16G | CGAGUAAGCUAAAAA**G**CAAUU |
| siPrnp105(T10)-17G | CGAGUAAGCUAAAAAC**G**AAUU |
| siPrnp178(A9)-5C | UUGU**C**CACAACUGCGUCAAUU |
| siPrnp178(A9)-6G | UUGUG**G**ACAACUGCGUCAAUU |
| siPrnp178(A9)-12A | UUGUGCACAAC**A**GCGUCAAUU |
| siPrnp178(A9)-13C | UUGUGCACAACU**C**CGUCAAUU |
| siPrnp178(A9)-14G | UUGUGCACAACUG**G**GUCAAUU |
| siPrnp178(A9)-15C | UUGUGCACAACUGC**C**UCAAUU |
| siPrnp178(A9)-16A | UUGUGCACAACUGCG**A**CAAUU |
| siPrnp178(A9)-17G | UUGUGCACAACUGCGU**G**AAUU |

Sense (passenger)-strand siRNAs containing 2-nt ribo-uridine (UU) 3’ overhangs are indicated. Mismatched nucleotides are highlighted.
