# Supplementary material for: Enhancement of Allele Discrimination by Introduction of Nucleotide Mismatches into siRNA in Allele-Specific Gene Silencing by RNAi
Source: PLoS One. 2008 May 21;3(5):e2248. doi: 10.1371/journal.pone.0002248 (PMC2373929; doi:10.1371/journal.pone.0002248)
Supplement: Table S3 — (0.03 MB DOC) [file pone.0002248.s006.doc]

Table s3. Synthetic DNA oligonucleotides

| Name | Seq. (5’---------------------3’) |
| --- | --- |
| ss-WT-Prnp (102,105) | CTAGAGTCAGTGGAACAAGCCGAGTAAGCCAAAAACCAACATGAATTCGC |
| as-WT-Prnp (102,105) | GGCCGCGAATTCATGTTGGTTTTTGGCTTACTCGGCTTGTTCCACTGACT |
| ss-Prnp(P102L) | CTAGAGTCAGTGGAACAAGCTGAGTAAGCCAAAAACCAACATGAATTCGC |
| as-Prnp(P102L) | GGCCGCGAATTCATGTTGGTTTTTGGCTTACTCAGCTTGTTCCACTGACT |
| ss-Prnp(P105L) | CTAGAGTCAGTGGAACAAGCCGAGTAAGCTAAAAACCAACATGAATTCGC |
| as-Prnp(P105L) | GGCCGCGAATTCATGTTGGTTTTTAGCTTACTCGGCTTGTTCCACTGACT |
| ss-WT-Prnp(D178N) | CTAGAGAACAACTTTGTGCACGACTGCGTCAATATCACAAGCTTGC |
| as-WT-Prnp(D178N) | GGCCGCAAGCTTGTGATATTGACGCAGTCGTGCACAAAGTTGTTCT |
| ss-Prnp(D178N) | CTAGAGAACAACTTTGTGCACAACTGCGTCAATATCACAAGCTTGC |
| as-Prnp(D178N) | GGCCGCAAGCTTGTGATATTGACGCAGTTGTGCACAAAGTTGTTCT |
| ss-HOXB8 3'UTR | TCGAGTCTCCCAACAACATGAAACTGCCTATTCACTTACTAGT |
| as-HOXB8 3'UTR | ACTAGTAAGTGAATAGGCAGTTTCATGTTGTTGGGAGAC |

‘ss-’ and ‘as-’ prefixed to the names represent the sense- and antisense-strand sequences, respectively.
