# Supplementary material for: Enhancement of Allele Discrimination by Introduction of Nucleotide Mismatches into siRNA in Allele-Specific Gene Silencing by RNAi
Source: PLoS One. 2008 May 21;3(5):e2248. doi: 10.1371/journal.pone.0002248 (PMC2373929; doi:10.1371/journal.pone.0002248)
Supplement: Table S4 — (0.03 MB DOC) [file pone.0002248.s007.doc]

Table s4. Synthetic DNA oligonucleotides for construction of shRNA expression vectors

| Name | Seq. (5’---------------------3’) |
| --- | --- |
| ss-shCont | GATCCGTTCTCCGAACGTGTCACGTTCTTGCTTCCTGTCACAAGAACGTGACACGTTCGGAGAACTTTTTTCTCGAGGC |
| as-shCont | GGCCGCCTCGAGAAAAAAGTTCTCCGAACGTGTCACGTTCTTGTGACAGGAAGCAAGAACGTGACACGTTCGGAGAACG |
| ss-sh102(T9) | GATCCGGAACAAGCTGAGTAAGCCACTTGCTTCCTGTCACAAGTGGCTTACTCAGCTTGTTCCTTTTTTCTCGAGGC |
| as-sh102(T9) | GGCCGCCTCGAGAAAAAAGGAACAAGCTGAGTAAGCCACTTGTGACAGGAAGCAAGTGGCTTACTCAGCTTGTTCCG |
| ss-sh102(T9)-12C | GATCCGGAACAAGCTGACTAAGCCACTTGCTTCCTGTCACAAGTGGCTTAGTCAGCTTGTTCCTTTTTTCTCGAGGC |
| as-sh102(T9)-12C | GGCCGCCTCGAGAAAAAAGGAACAAGCTGACTAAGCCACTTGTGACAGGAAGCAAGTGGCTTAGTCAGCTTGTTCCG |
| ss-sh102(T9)-13A | GATCCGGAACAAGCTGAGAAAGCCACTTGCTTCCTGTCACAAGTGGCTTTCTCAGCTTGTTCCTTTTTTCTCGAGGC |
| as-sh102(T9)-13A | GGCCGCCTCGAGAAAAAAGGAACAAGCTGAGAAAGCCACTTGTGACAGGAAGCAAGTGGCTTTCTCAGCTTGTTCCG |
| ss-sh102(T9)-14U | GATCCGGAACAAGCTGAGTTAGCCACTTGCTTCCTGTCACAAGTGGCTAACTCAGCTTGTTCCTTTTTTCTCGAGGC |
| as-sh102(T9)-14U | GGCCGCCTCGAGAAAAAAGGAACAAGCTGAGTTAGCCACTTGTGACAGGAAGCAAGTGGCTAACTCAGCTTGTTCCG |
| ss-sh102(T9)-15U | GATCCGGAACAAGCTGAGTATGCCACTTGCTTCCTGTCACAAGTGGCATACTCAGCTTGTTCCTTTTTTCTCGAGGC |
| as-sh102(T9)-15U | GGCCGCCTCGAGAAAAAAGGAACAAGCTGAGTATGCCACTTGTGACAGGAAGCAAGTGGCATACTCAGCTTGTTCCG |

‘ss-’ and ‘as-’ prefixed to the names represent the sense- and antisense-strand sequences, respectively.
