# Supplementary material for: Enhancement of Allele Discrimination by Introduction of Nucleotide Mismatches into siRNA in Allele-Specific Gene Silencing by RNAi
Source: PLoS One. 2008 May 21;3(5):e2248. doi: 10.1371/journal.pone.0002248 (PMC2373929; doi:10.1371/journal.pone.0002248)
Supplement: Table S5 — (0.04 MB DOC) [file pone.0002248.s008.doc]

Table s5. Synthetic forked siRNA duplexes against London and Swedish-type *APP* variants

| Name | Seq. (5'-------3') |
| --- | --- |
| F-siAPP(A8)-ss | AGUGAUCAUCAUCACCUAU |
| siAPP(A8)-as | CAAGGUGAUGAUGAUCACUUU |
| F-siAPP(A10)-ss | ACAGUGAUCAUCAUCACUA |
| siAPP(A10)-as | AGGUGAUGAUGAUCACUGUUU |
| F-siAPP(A11)-ss | GACAGUGAUCAUCAUCAUU |
| siAPP(A11)-as | GGUGAUGAUGAUCACUGUCUU |
| F-siAPP(T8)-ss | AGUGAUCUUCAUCACCUAU |
| siAPP(T8)-as | CAAGGUGAUGAAGAUCACUUU |
| F-siAPP(T10)-ss | ACAGUGAUCUUCAUCACUA |
| siAPP(T10)-as | AGGUGAUGAAGAUCACUGUUU |
| F-siAPP(T11)-ss | GACAGUGAUCUUCAUCAUU |
| siAPP(T11)-as | GGUGAUGAAGAUCACUGUCUU |
| F-siAPP(G8)-ss | GUGAUCGGCAUCACCUUUU |
| siAPP(G8)-as | CCAAGGUGAUGCCGAUCACUU |
| F-siAPP(G9)-ss | AGUGAUCGGCAUCACCUAU |
| siAPP(G9)-as | CAAGGUGAUGCCGAUCACUUU |
| F-siAPP(G10)-ss | CAGUGAUCGGCAUCACCAA |
| siAPP(G10)-as | AAGGUGAUGCCGAUCACUGUU |
| F-siAPP(G11)-ss | ACAGUGAUCGGCAUCACUA |
| siAPP(G11)-as | AGGUGAUGCCGAUCACUGUUU |
| F-siAPP(G12)-ss | GACAGUGAUCGGCAUCAUU |
| siAPP(G12)-as | GGUGAUGCCGAUCACUGUCUU |
| F-siAPP(T12/C13)-ss | UCUGAAGUGAAUCUGGAUUA |
| siAPP(T12/C13)-as | GCAUCCAGAUUCACUUCAGAUU |

The 3’-ends of forked sense-strand siRNA element (-ss) contain two nucleotide mismatches against the antisense-strand siRNA element (-as). The annealed fork-siRNA duplexes (F-siRNA duplexes) are schematically shown in Figure 4A.
