# Supplementary material for: Enhancement of Allele Discrimination by Introduction of Nucleotide Mismatches into siRNA in Allele-Specific Gene Silencing by RNAi
Source: PLoS One. 2008 May 21;3(5):e2248. doi: 10.1371/journal.pone.0002248 (PMC2373929; doi:10.1371/journal.pone.0002248)
Supplement: Table S6 — (0.03 MB DOC) [file pone.0002248.s009.doc]

Table s6. Synthetic miRNA duplexes

| miR-196a | 5' -**UAGGUAGUUUCAUGUUGUUGGG** |
| --- | --- |
|  | GAAUUCAUCAAAGUACAACAAC- 5' |
| miR-196b | 5' -**UAGGUAGUUUCCUGUUGUUGGG** |
|  | GAAUUCAUCAAAGGACAACAAC- 5' |

The miR-196a duplexes were chemically synthesized according to the previous study [31]. Matured miRNA sequences are shown in bold.
