# Supplementary material for: Enhancement of Allele Discrimination by Introduction of Nucleotide Mismatches into siRNA in Allele-Specific Gene Silencing by RNAi
Source: PLoS One. 2008 May 21;3(5):e2248. doi: 10.1371/journal.pone.0002248 (PMC2373929; doi:10.1371/journal.pone.0002248)
Supplement: Table S7 — (0.11 MB DOC) [file pone.0002248.s010.doc]

Table s7. Nucleotide sequences of siRNAs conferring less (A) and potent (B) RNAi activity

| (A) | **1** | **2** | **3** | **4** | **5** | **6** | **7** | **8** | **9** | **10** | **11** | **12** | **13** | **14** | **15** | **16** | **17** | **18** | **19** | **RNAi activities** |
| --- | --- | --- | --- | --- | --- | --- | --- | --- | --- | --- | --- | --- | --- | --- | --- | --- | --- | --- | --- | --- |
| **siPrnp102(T11)** | **U** | **G** | **G** | **A** | **A** | **C** | **A** | **A** | **G** | **C** | **U** | **G** | **A** | **G** | **U** | **A** | **A** | **G** | **C** | **-** |
| **siAPP(A8)** | **A** | **G** | **U** | **G** | **A** | **U** | **C** | **A** | **U** | **C** | **A** | **U** | **C** | **A** | **C** | **C** | **U** | **U** | **G** | **-** |
| **siAPP(A10)** | **A** | **C** | **A** | **G** | **U** | **G** | **A** | **U** | **C** | **A** | **U** | **C** | **A** | **U** | **C** | **A** | **C** | **C** | **U** | **-** |
| **siAPP(A11)** | **G** | **A** | **C** | **A** | **G** | **U** | **G** | **A** | **U** | **C** | **A** | **U** | **C** | **A** | **U** | **C** | **A** | **C** | **C** | **-** |
| **siAPP(T8)** | **A** | **G** | **U** | **G** | **A** | **U** | **C** | **U** | **U** | **C** | **A** | **U** | **C** | **A** | **C** | **C** | **U** | **U** | **G** | **-** |
| **siAPP(T11)** | **G** | **A** | **C** | **A** | **G** | **U** | **G** | **A** | **U** | **C** | **U** | **U** | **C** | **A** | **U** | **C** | **A** | **C** | **C** | **-** |
| **siAPP(G8)** | **G** | **U** | **G** | **A** | **U** | **C** | **G** | **G** | **C** | **A** | **U** | **C** | **A** | **C** | **C** | **U** | **U** | **G** | **G** | **-** |
| **siAPP(G9)** | **A** | **G** | **U** | **G** | **A** | **U** | **C** | **G** | **G** | **C** | **A** | **U** | **C** | **A** | **C** | **C** | **U** | **U** | **G** | **-** |
| **siAPP(G11)** | **A** | **C** | **A** | **G** | **U** | **G** | **A** | **U** | **C** | **G** | **G** | **C** | **A** | **U** | **C** | **A** | **C** | **C** | **U** | **-** |
| **siAPP(G12)** | **G** | **A** | **C** | **A** | **G** | **U** | **G** | **A** | **U** | **C** | **G** | **G** | **C** | **A** | **U** | **C** | **A** | **C** | **C** | **-** |

| (B) | **1** | **2** | **3** | **4** | **5** | **6** | **7** | **8** | **9** | **10** | **11** | **12** | **13** | **14** | **15** | **16** | **17** | **18** | **19** | **RNAi activities** |
| --- | --- | --- | --- | --- | --- | --- | --- | --- | --- | --- | --- | --- | --- | --- | --- | --- | --- | --- | --- | --- |
| **siPrnp102(T9)** | **G** | **A** | **A** | **C** | **A** | **A** | **G** | **C** | **U** | **G** | **A** | **G** | **U** | **A** | **A** | **G** | **C** | **C** | **A** | **++** |
| **siPrnp102(T10)** | **G** | **G** | **A** | **A** | **C** | **A** | **A** | **G** | **C** | **U** | **G** | **A** | **G** | **U** | **A** | **A** | **G** | **C** | **C** | **++** |
| **siPrnp105(T10)** | **C** | **G** | **A** | **G** | **U** | **A** | **A** | **G** | **C** | **U** | **A** | **A** | **A** | **A** | **A** | **C** | **C** | **A** | **A** | **++** |
| **siPrnp105(T11)** | **C** | **C** | **G** | **A** | **G** | **U** | **A** | **A** | **G** | **C** | **U** | **A** | **A** | **A** | **A** | **A** | **C** | **C** | **A** | **++** |
| **siPrnp105(T12)** | **G** | **C** | **C** | **G** | **A** | **G** | **U** | **A** | **A** | **G** | **C** | **U** | **A** | **A** | **A** | **A** | **A** | **C** | **C** | **++** |
| **siPrnp178(A7)** | **G** | **U** | **G** | **C** | **A** | **C** | **A** | **A** | **C** | **U** | **G** | **C** | **G** | **U** | **C** | **A** | **A** | **U** | **A** | **++** |
| **siPrnp178(A8)** | **U** | **G** | **U** | **G** | **C** | **A** | **C** | **A** | **A** | **C** | **U** | **G** | **C** | **G** | **U** | **C** | **A** | **A** | **U** | **++** |
| **siPrnp178(A9)** | **U** | **U** | **G** | **U** | **G** | **C** | **A** | **C** | **A** | **A** | **C** | **U** | **G** | **C** | **G** | **U** | **C** | **A** | **A** | **++** |
| **siPrnp178(A10)** | **U** | **U** | **U** | **G** | **U** | **G** | **C** | **A** | **C** | **A** | **A** | **C** | **U** | **G** | **C** | **G** | **U** | **C** | **A** | **++** |
| **siPrnp178(A11)** | **C** | **U** | **U** | **U** | **G** | **U** | **G** | **C** | **A** | **C** | **A** | **A** | **C** | **U** | **G** | **C** | **G** | **U** | **C** | **++** |
| **siPrnp178(A12)** | **A** | **C** | **U** | **U** | **U** | **G** | **U** | **G** | **C** | **A** | **C** | **A** | **A** | **C** | **U** | **G** | **C** | **G** | **U** | **++** |
| **siPrnp105(T9)** | **G** | **A** | **G** | **U** | **A** | **A** | **G** | **C** | **U** | **A** | **A** | **A** | **A** | **A** | **C** | **C** | **A** | **A** | **C** | **ASP** |

siRNAs conferring little or no RNAi activity (-) and strong RNAi activity against both mutant and wild-type alleles (++) and also allele-specific RNAi (ASP) are grouped and aligned in A and B, respectively. The previous data of assessment of siRNAs [17] were also used. The nucleotide sequences of assessed siRNAs are aligned such that the 5’-ends of the sense-stranded siRNA elements can be matched. Nucleotide potions form the 5’-end of the sequence are indicated. G or C residues are indicated in gray background.
